# CpG island composition differences are a source of gene expression noise indicative of promoter responsiveness

**DOI:** 10.1186/s13059-018-1461-x

**Published:** 2018-06-26

**Authors:** Michael D. Morgan, John C. Marioni

**Affiliations:** 1 Wellcome Trust Sanger Institute, Wellcome Genome Campus, Hinxton, Cambridge, CB10 1SA UK; 2 Cancer Research UK Cambridge Institute, University of Cambridge, Robinson Way, Li Ka Shing Centre, Cambridge, CB2 0RE UK; 3 European Molecular Biology Laboratory, European Bioinformatics Institute (EMBL-EBI), Wellcome Genome Campus, Hinxton, Cambridge, CB10 1SD UK

**Keywords:** Gene expression noise, Single cell, Promoter response

## Abstract

**Background:**

Population phenotypic variation can arise from genetic differences between individuals, or from cellular heterogeneity in an isogenic group of cells or organisms. The emergence of gene expression differences between genetically identical cells is referred to as gene expression noise, the sources of which are not well understood.

**Results:**

In this work, by studying gene expression noise between multiple cell lineages and mammalian species, we find consistent evidence of a role for CpG islands as sources of gene expression noise. Variation in noise among CpG island promoters can be partially attributed to differences in island size, in which short islands have noisier gene expression. Building on these findings, we investigate the potential for short CpG islands to act as fast response elements to environmental stimuli. Specifically, we find that these islands are enriched amongst primary response genes in SWI/SNF-independent stimuli, suggesting that expression noise is an indicator of promoter responsiveness.

**Conclusions:**

Thus, through the integration of single-cell RNA expression profiling, chromatin landscape and temporal gene expression dynamics, we have uncovered a role for short CpG island promoters as fast response elements.

**Electronic supplementary material:**

The online version of this article (10.1186/s13059-018-1461-x) contains supplementary material, which is available to authorized users.

## Background

Variability in gene expression across an isogenic population of cells has garnered significant interest over the past decade and a half. Initial studies of variability in gene expression, henceforth referred to as gene expression noise, from single promoters [1, 2] and simple gene regulatory circuits [3] have motivated work in pro- and eukaryote systems to characterise and investigate both the sources and consequences of this noise. The extent of noise within vertebrate, and more specifically mammalian, systems has received less attention [4]. In part this is due to the simplicity and elegance of organisms that exist primarily in a single-cell state for much of their natural lives, but also the ease with which these organisms and systems can be manipulated to alter gene expression noise. This is especially important for understanding the drivers of expression noise, as well as its consequences at the biochemical, cellular and physiological levels.

In yeast, the presence of a TATA-box motif in the core promoter is linked with greater noise, which is associated with differences in nucleosome occupancy and pre-initiation complex dynamics [5, 6]. Examination of mammalian promoter features that influence individual-to-individual variability have highlighted a number of chromatin modifications linked to differential variability [7]. Likewise, the integration of single-cell expression profiling with bulk chromatin modification data in embryonic cells has highlighted a role for differences in chromatin modifications between genes as a possible source of gene expression noise [8–10]. Specifically, both Kar et al. [8] and Faure et al. [10] show that promoter chromatin modifications are related to gene expression noise differences between promoters, whilst Wu et al. find that gene body chromatin methylation, specifically H3K79me3, is associated with differential noise [9]. Additionally, both [8] and [10] demonstrate that promoters that are simultaneously marked with opposing chromatin states (repressive H3K27me3 and activation H3K4me3), so-called bivalent promoters [11], are associated with greater gene expression noise in bulk populations of embryonic stem cells. This may arise due to heterogeneity in the chromatin state across the populations of cells studied or due to opposing chromatin modification-associated activity at these promoters [10].

We hypothesise that there are a number of promoter features that may influence the expression noise of a particular gene. To understand the shared features of promoters, we investigated potential sources and causes of gene expression noise at a genome-wide scale. We find that CpG island promoters are associated with less gene expression noise than their non-CpG island counterparts, and that the characteristic features of CpG islands, e.g. polycomb repressor complex (PRC) and trithorax group (TrxG) chromatin modifications and CpG island size, contribute to differential noise between promoters. Within CpG island promoters, there remain extensive differences in gene expression noise, which is anti-correlated with CpG island size. We corroborate recent findings showing that bivalent promoters in mouse embryonic stem cells (mESCs) are the noisiest promoters [8, 10]. Expanding on the work by Faure et al. [10], we propose that the increased noise from short CpG islands is an indication of promoter dynamics. Specifically, we investigate whether short CpG islands act as agile response elements for switch/sucrose non-fermentable complex (SWI/SNF)-independent primary response genes. Analysis of time-series expression profiles from human and mouse studies suggests that under different stimuli, genes with short CpG islands respond earlier than those with longer CpG islands and that highly variable genes in unstimulated cells form part of the early response.

## Results

### Genomic sources of gene expression noise

We considered different categories of genomic features involved in gene expression regulation, to capture generic features of gene promoter architecture that influence expression dynamics (Additional file 1: Table S1). These features can be sub-divided into static and dynamic types. Static features are invariant between cells, whilst dynamic features can vary between cells depending upon their lineage, type or state.

**Fig. 1:**
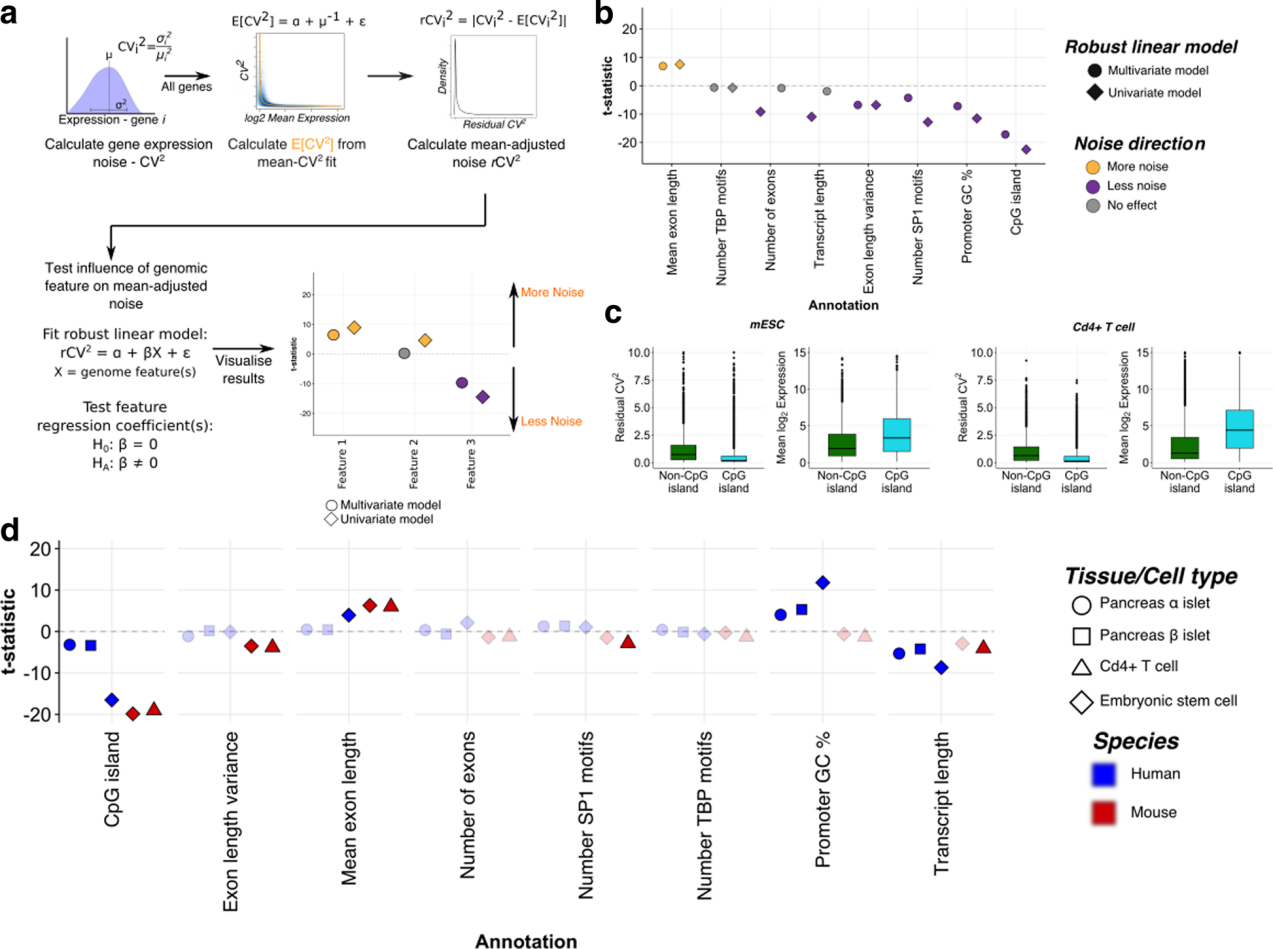
Scan for promoter features associated with gene expression noise. **a** Gene expression noise is measured using the squared coefficient of variation (CV^2^) for each gene (subscript *i*). The relationship between the mean and CV^2^, illustrated by the smoothed scatter plot (blue cloud), is accounted for by calculating an expected value for the gene expression noise for each gene (*E*[CV^2^]), shown by the orange line. The residual coefficient of variation *r*CV^2^, or the mean-adjusted gene expression noise, is calculated as the absolute deviation of the observed CV^2^ from its expected value, shown in the kernel density plot (right panel). The influence of genomic features is tested by fitting a robust linear model to prevent outlier points biasing our results, using the *r*CV^2^ as the dependent variable. Each feature is regressed on *r*CV^2^ individually (univariate model: *X* is a vector of values representing the genomic feature; multivariate model: *X* denotes a matrix where each column is a genomic feature and the rows are genes). The statistical significance is determined by testing the null hypothesis that the genomic feature regression coefficient, *β*, is equal to 0 using a *t*-test. Univariate and multivariate model fitting results are visualised side-by-side for all genomic features, as in the toy example (bottom right panel). Points above 0 (orange) are associated with greater noise, whilst those below the line are associated with lower noise (purple). **b** Static promoter features were regressed on expression noise (*r*CV^2^) in mESCs and mouse Cd4+ T cells (Additional file 2: Figure S1). Each point represents a genomic element in either a univariate (diamond) or multivariate (circle) robust linear regression model. Grey points denote features in which there is insufficient statistical evidence to reject the null hypothesis of no association with expression noise (*p*<0.05). **c** Examples of how gene expression noise differs between CGI and non-CGI promoters in both mouse embryonic stem cells and Cd4+ T cells. The *y*-axes in the plot of *r*CV^2^ are truncated for clarity. **d** Genomic features across multiple cell-type lineages and between mouse and human that are consistently associated with transcriptional noise in a multivariate robust linear model. Cell types are denoted by different symbols; human- and mouse-derived cells are delineated by colour (blue for human and red for mouse). Transparent points are those where there is insufficient statistical evidence to reject the null hypothesis (*p*<0.05). The *y*-axis ranges in (**b**) and (**d**) are truncated for clarity. mESC mouse embryonic stem cell

To screen for genomic elements that influence gene expression noise at the global level, we model the relative noise of each gene as the squared coefficient of variation (CV^2^). There is an inherent relationship between the mean and the variance for data generated by a time-interval counting process, e.g. a Poisson process. This must be accounted for so that any correlations with gene expression noise can be disentangled from correlations with the average expression across cells. Thus, we fit a reciprocal relationship between the mean log2 expression values and CV^2^, parametrised as in Brennecke et al. [12] (Fig. 1a). We call this mean-adjusted measure the residual CV^2^ (*r*CV^2^). To find the influence of each genomic feature on the mean-adjusted noise, we regress each feature on the *r*CV^2^ (across all genes), as illustrated in Fig. 1a.

Using single-cell RNA-seq measurements from mESCs [13], we first explored which genomic features could underpin differences in (mean independent) expression noise across cells. Using the approach described (‘Methods’; Fig. 1a), we evaluated the effect of each promoter feature on gene expression noise (Fig. 1b–d). This included a multivariate robust linear model with all of the genomic features to test for linear independence between possible related features (Fig. 1b). To replicate the observed effects independently and to test their generalisability, we performed the same analysis using data derived from mouse Cd4+ naive T cells [14] (Additional file 2: Figure S1).

Unlike previous reports on *Saccharomyces cerevisiae* and mESCs, we do not observe a consistent correlation between predicted TATA box binding protein (TBP) motifs and differences in expression noise [10, 15] (Fig. 1b). In this study, we consider that the promoter encompasses a 1.5-kb region, whilst previous studies on TATA boxes and TBP binding have restricted their analysis to core promoter regions (∼200 bp) centred on the transcriptional start site. Using the same definition of TATA-box promoters as in [10, 16], we find that TATA-box promoters are associated with greater gene expression noise in our univariate, but not the multivariate, robust regression model (Additional file 2: Figure S2). Thus, this discrepancy arises due to differences between relying solely on predicted TBP motifs and more comprehensive promoter classifications, rather than the size of the promoter region per se.

We find in the univariate case that gene structure (i.e. transcript length, number of exons and mean exon length) has a relatively large influence on noise (Fig. 1b, circles). With the exception of mean exon length, these effects are consistently captured by other variables related to gene structure in both mESCs and Cd4+ T cells. Interestingly, we find that promoters with an overlapping CpG island are on average less variable than their non-CpG island counterparts (Fig. 1 and Additional file 2: Figure S1), concordant with a recent report by Faure et al. [10].

As we wish to understand the general features of mammalian promoters that influence their noise, we extended our analysis to several human cell types (Additional file 3: Table S2). In accordance with our observations in mouse, we observe that CpG islands are consistently associated with lower gene expression noise (Fig. 1d). The extent to which CpG islands are correlated with gene expression noise varies between cell types and between species. This may represent biological differences between developmental and evolutionary lineages or technical and experimental differences between studies. The data sets used in our analysis are all generated using the SMART-seq(2) chemistry [17, 18], and thus, may be susceptible to technical noise arising from fragment duplication. To test whether our results are affected by this potential bias, we also performed the same analysis using single-cell expression profiles from mESCs cultured in serum + leukaemia inhibitory factor, generated using unique molecular identifiers [19]. We find that CpG islands remain associated with lower expression noise, suggesting that this correlation does not arise due to shared technical sources of variation in single-cell RNA-seq experiments (Additional file 2: Figure S3). Subsequently, we can confidently conclude that the relationship between differential noise and CpG island and non-CpG island promoters is a feature of mammalian genomes separated by ∼80 million years of evolution.

### Characteristics of CpG islands associated with expression noise

Although genes with CpG island promoters are systematically less noisy than genes without a CpG island, there is still considerable variability in expression levels *among* CpG island genes (Fig. 1c, black outlier points). This raises the question of whether the characteristics of specific CpG islands also contribute to gene expression noise, which to our knowledge has not been previously addressed. We selected features of CpG islands to test for association with gene expression noise, such as CpG island size and the number of predicted SP1 binding motifs. Several features of CpG islands are highly correlated, e.g. CpG island size and CpG dinucleotide ratio (Additional file 2: Figure S4). For this reason, we selected CpG island size as a characteristic measure of CpG islands, as it has a more intuitive interpretation and has been linked with potential functional differences between genes [20].

**Fig. 2:**
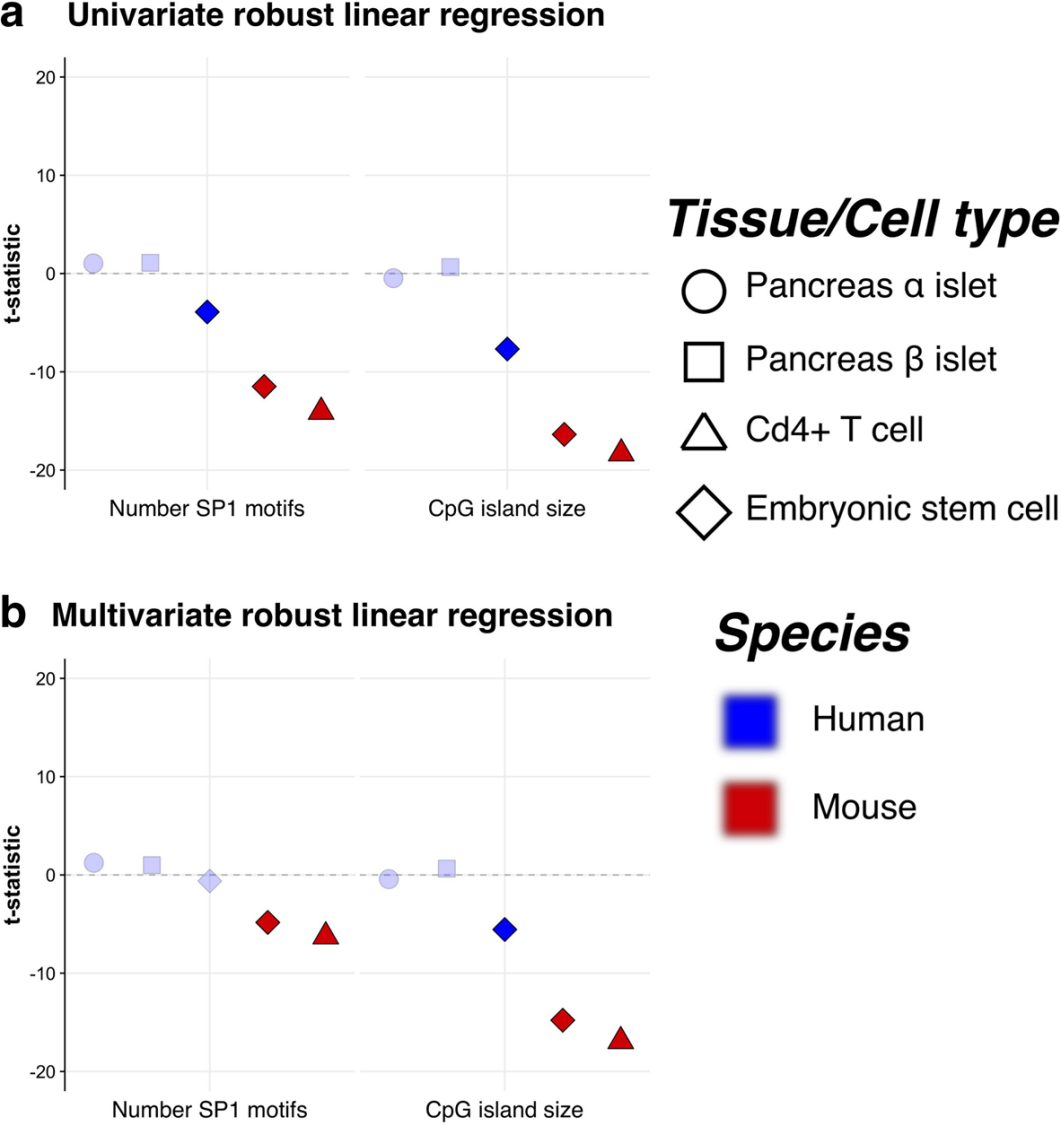
Robust linear regression analysis of CpG island features across cell types. **a** Univariate robust linear regression analysis of CpG island features across cell types associated with expression noise. **b** Multivariate robust linear regression analysis of CpG island features across cell types associated with noise. For both human and mouse cell types, robust linear models were fitted to *r*CV^2^ for all genes with an associated CpG island. Points that fall above the horizontal line are associated with greater noise, whilst those below are associated with lower. Features for which there is insufficient statistical evidence to reject the null hypothesis of no association with gene expression noise have a lighter shading. Symbols represent the different cell types (circles for *α*-islets, squares for *β*-islets, triangles for naive Cd4+ T cells and diamonds for embryonic stem cells). Human and mouse cell types are denoted by colours (blue for human and red for mouse)

We tested each feature of CpG islands individually across cell types. This univariate analysis found that in both human and mESCs, and murine Cd4 + T cells, CpG island size and the number of SP1 binding sites are individually associated with lower transcriptional noise (Fig. 2a). However, in human pancreatic *α*- and *β*-islet cells, CpG island size is generally not associated with differences in expression noise (Fig. 2a). To test whether the partial correlation between SP1 binding motifs and CpG island size influences gene expression noise independently, we fitted a multivariate robust linear model that included both features. We find that CpG island size and the number of predicted SP1 binding motifs are linearly independent in both mouse cell types, but not in human pancreas cells. The discordance between pancreatic islet cells and other cell types may be indicative of lineage-specific effects that alter the relationship between genomic features and gene expression noise. Applying the same model and analysis to additional single-cell pancreatic islet expression profiles gives similar results (Additional file 2: Figure S5). Overall, these results indicate that variation in CpG island size is consistently correlated with gene expression noise amongst different gene promoters independent of species.

### The epigenetic landscape is correlated with expression noise

We wish to understand the mechanism that leads to differences in gene expression noise among CpG islands. The principal difference between CpG island and non-CpG island promoters is how their transcription is repressed or modulated. Non-CpG island promoters maintain transcription repression by cytosine methylation at CpG dinucleotides [21], though recent evidence demonstrates that CpG methylation alone cannot induce repression [22]. CpG islands are constitutively unmethylated regions, despite a high CpG density that would attract active DNA methyltransferases [21]. These unmethylated CpG dinucleotides provide a platform for binding by proteins that form part of the PRC and TrxG, via their CxxC domains [23, 24]. Thus, transcription from CpG island promoters is primarily regulated by chromatin histone modifications, in the absence of direct modification of DNA, such as cytosine methylation.

To understand how CpG islands contribute to noise in gene expression, we combined chromatin histone modification data associated with transcriptional activation and repression (H3K4me3 and H3K27me3 ChIP-seq; see ‘Methods’) on bulk mESCs with single-cell RNA-seq expression profiling. Previous work has highlighted the enrichment of genes involved in developmental processes amongst promoters with long CpG islands [20, 25]. One explanation for the observed relationship between CpG island size and decreasing expression noise (Fig. 2a), therefore, might be that developmental genes need to have more highly regulated expression. However, after removing >900 developmentally-associated genes, we find that the anti-correlation between CpG island features and expression noise remains, suggesting there is an alternative explanation (Additional file 2: Figure S6).

**Fig. 3:**
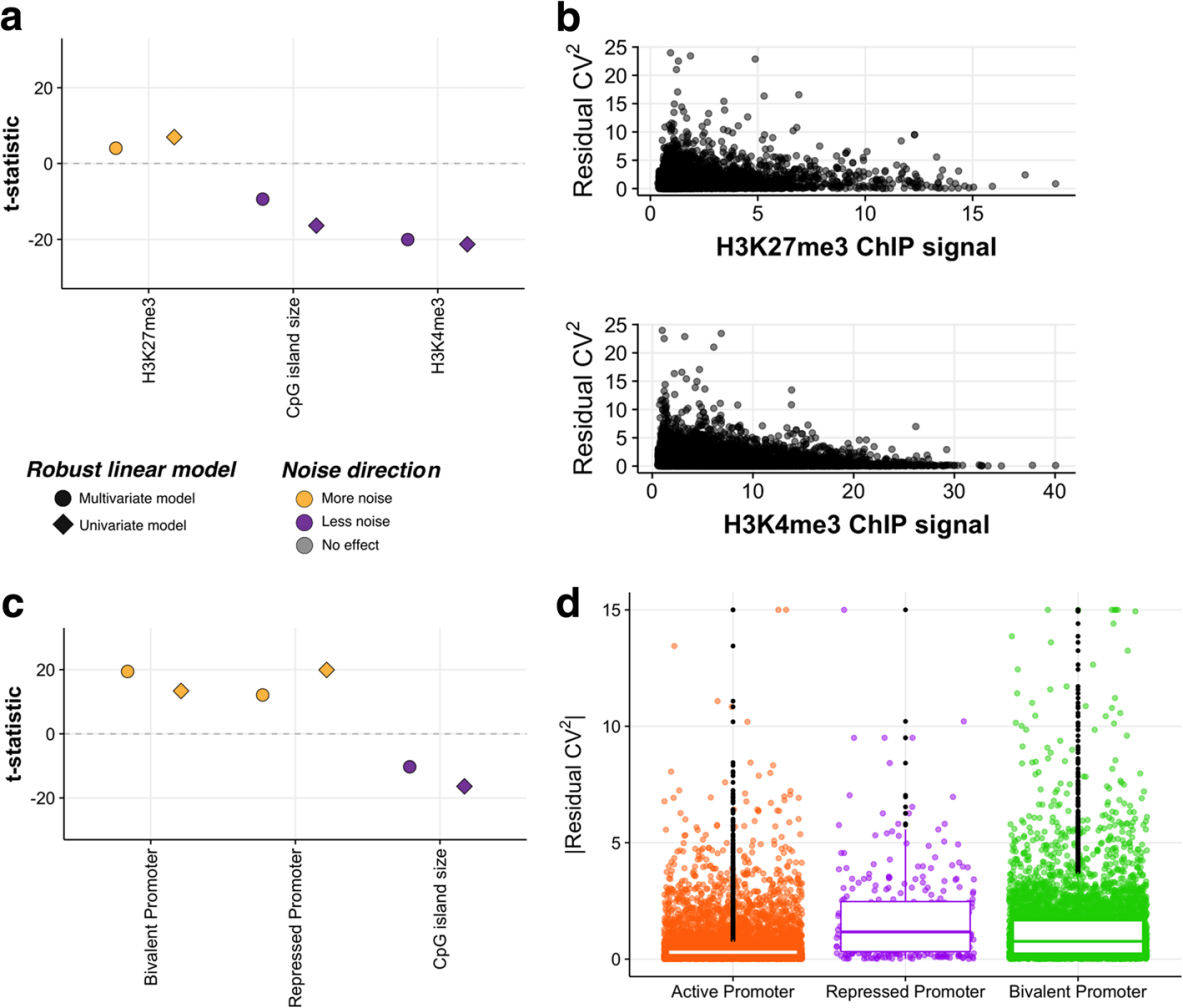
Promoter chromatin modifications influence expression noise in mESCs. **a** Robust linear regression model results, modelling the influence of promoter chromatin landscape and CpG island size on expression noise. Points below the horizontal dotted line are correlated with lower noise (purple), whilst those above are associated with greater noise (orange). **b** Promoter chromatin modifications (fold enrichment for ChIP/input) for repressive (H3K27me3; top panel) and active (H3K4me3; bottom panel) expression are negatively correlated with expression noise. **c** Bivalent promoters are the noisiest promoters. Robust linear regression model results of expression noise, jointly fitting promoter category relative to the *repressed* promoters, with CpG island size. **d** The relationship between promoter chromatin modification category and *r*CV^2^. Overlaid box plots represent the median *r*CV^2^ for each category, with lower and upper quartiles. Individual points lying outside the interquartile range are plotted as black points. The *y*-axis is truncated at [0, 15] for clarity. mESC mouse embryonic stem cell

If CpG islands directly represent a platform for chromatin modifiers that bind to unmethylated CpGs, the size of a CpG island may influence the amount of bound PRC or chromatin modification laid down at a locus. If this is the case, then the amount of H3K4me3 and H3K27me3 would be anti-correlated with gene expression noise. Indeed, we find in mESCs that CGI size and H3K4me3 are individually correlated with lower expression noise as expected (Fig. 3a), when considering only CpG island promoters. However, the opposite pattern is observed for H3K27me3. The promoters at which transcription is truly repressed would not produce any transcripts; thus, the observed association of greater noise with H3K27me3 may represent bivalent promoters [11]. Bivalent promoters are actively transcribed genes whose promoters are marked by both repressive and active chromatin modifications. The observed relationships between histone tail modifications and expression noise demonstrates that the greatest variability in gene expression noise between different promoters lies within regions with the lowest amount of modification (Fig. 3b). This may arise due to consistent differences between loci across all cells, i.e. all cells have a similar amount of H3 modifications at a given locus, or due to differences in the chromatin state between cells. This represents a limitation of integrating bulk-level chromatin modification with single-cell expression profiling. To resolve which mechanism explains the observed correlation would require per-locus chromatin modification information at single-cell resolution.

To investigate the potential for bivalency to explain the correlation between gene expression noise and CGI size, we categorised promoters as *repressed*, *active* or *bivalent* based on the combined H3K27me3 and H3K4me3 ChIP signal (Additional file 2: Figure S7). Consistent with recent reports [8, 10], our analysis reveals that bivalent promoters are highly variable, an effect that is not dependent on CpG island size (Fig. 3c).

### Short CpG islands provide response agility to stimulus

Our observations that CpG island size is associated with expression noise is not explained by the tendency for developmental genes to be associated with longer CpG islands (Additional file 2: Figure S6), nor by any correlation between CpG island size and bivalency. The necessity for tight regulation (i.e. a larger buffer against inappropriate stochastic activation) for genes involved in coordinated developmental programmes is well described. Whilst the gene expression noise of individual pluripotency and differentiation factors is associated with cellular plasticity and cell fate choice [26, 27], the execution of any particular developmental programme is highly coordinated and consistent, e.g. gastrulation, limb bud formation, etc. Mammalian CpG islands range in size from ∼200 bp to 10’s of kilobases and CpG island size is proportional between human and mouse orthologous genes (Spearman rank correlation = 0.51; Additional file 2: Figure S8). Whilst large islands may lead to tighter transcriptional regulation, it is not immediately clear why there is also concordance in the size of short CpG islands between homologous mouse and human genes.

Whilst we consider gene expression noise to arise due to influences from both *cis* and *trans* factors, the utility of gene expression noise remains unresolved. Previous work has described the benefits of gene expression noise in eukaryote systems [28], but often in relation to a single gene [28] or small gene regulatory network. If fluctuations in gene expression (i.e. noise) are detrimental to fitness, then buffering mechanisms may have evolved to reduce the impact of expression noise [29] or there could be some degree of tolerance to noise due to a cost/benefit trade-off. Thus, is the observed greater expression noise in small CpG islands indicative of a cost/benefit trade-off?

**Fig. 4:**
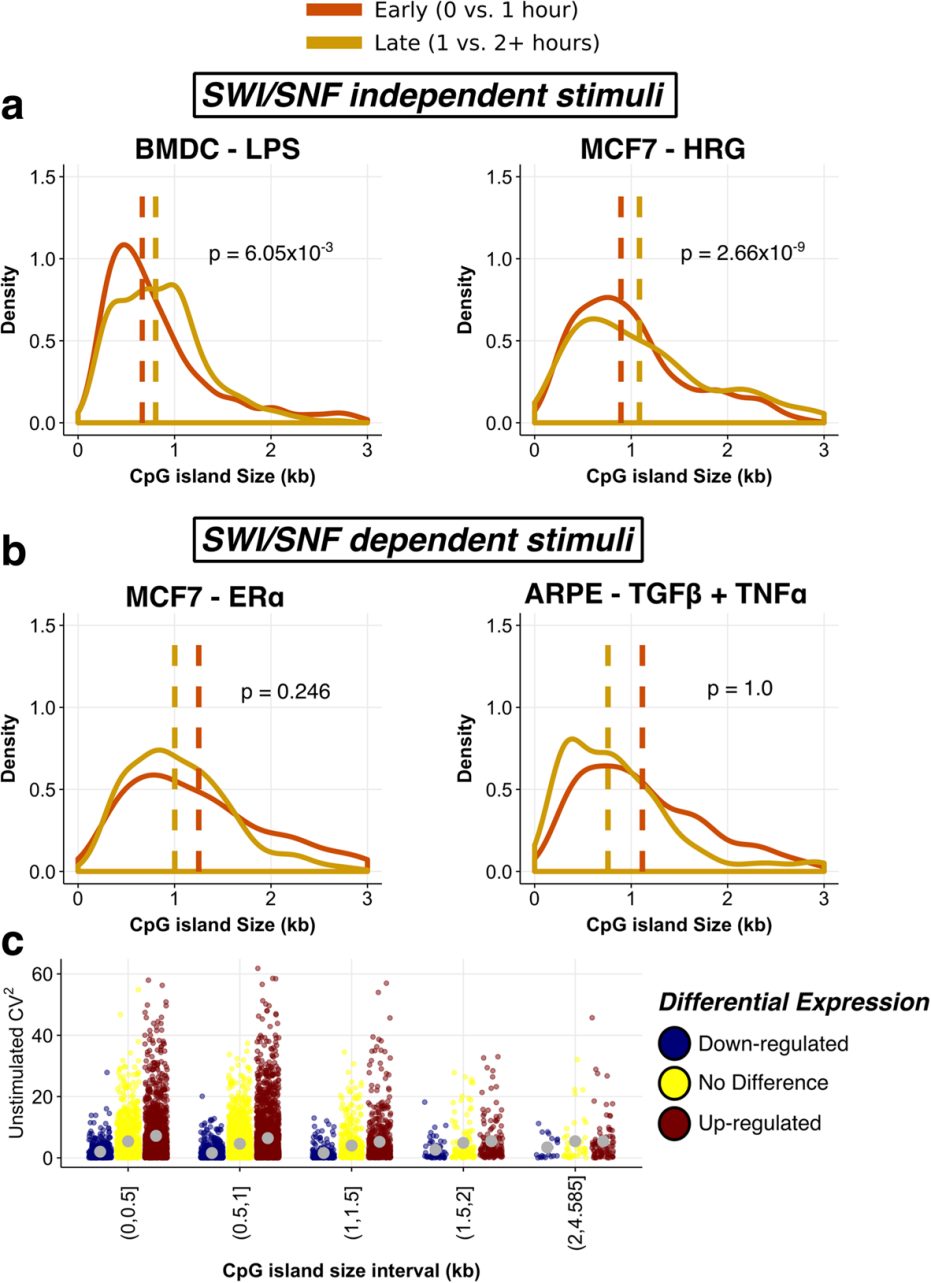
Short CpG islands are enriched in early response genes. **a** Murine LPS-stimulated BMDCs (left panel) and heregulin-stimulated human breast adenocarcinoma cells (right panel) demonstrate an enrichment for short CpG islands immediately post-stimulation compared to later time points. **b** Stimulation-specific enrichment for longer CpG islands post-stimulation in MCF7 breast adenocarcinoma (left) and retinal pigment epithelial cells (right). Plotted are the top 250 up-regulated genes with the largest log2-fold changes between time points (early, 0 vs 1 hr and late, 1 vs 2 hr). CpG island size is truncated at 3 kb for clarity. Vertical dashed lines denote the median CpG island length for the most up-regulated genes in the relevant time-point comparison (early is orange and late is yellow). **c** Highly variable genes in unstimulated BMDCs mark early response genes. Gene expression noise (CV^2^, *y*-axis) in unstimulated BMDCs across CpG island genes binned into 0.5-kb intervals (*x*-axis). Points are coloured by the direction of differential expression between unstimulated cells and 1-h post-stimulation (blue for down-regulated, yellow for no change and red for up-regulated). Filled grey circles represent the mean CV^2^ within each size category for differential expression. BMDC bone marrow-derived dendritic cell, ER *α* oestrogen receptor *α*, HRG heregulin, LPS lipopolysaccharide, SWI/SNF switch/sucrose non-fermentable complex

In aeronautical engineering, there is a trade-off between flight stability and agility, i.e. greater instability leads to greater flight responsiveness. We hypothesised that short CpG islands may act as fast response elements to environmental stimuli, i.e. they are enriched for immediate early genes (IEGs). To test this hypothesis, we collected publicly available time-series gene expression data for a number of different stimulation conditions and cell types (Additional file 4: Table S3). Under our hypothesis, we would expect there to be an enrichment of short CpG islands amongst the primary early response genes within the first hour of induction. Delayed and secondary response genes tend to lag by 2–4 h in induction [30]. In seven different cell types and stimuli, across both human and mouse tissues, we observe an enrichment for genes with short CpG islands being up-regulated immediately post-stimulation, compared to later time points (Fig. 4a). In mouse bone marrow-derived dendritic cells (BMDCs), early response genes are more likely to overlap or lie proximal to a short CpG island (binomial one-tailed *p*=6.05×10^−3^). To verify that this observed enrichment was not specific to LPS-stimulated dendritic cells, or to mouse, we tested for short CpG island enrichment in a time course of human breast adenocarcinoma cells stimulated with an ErbB3/4 ligand, heregulin. We found the same pattern of short CpG island enrichment immediately post-stimulation (binomial one-tailed *p*=2.66×10^−9^). Enrichment for short CpG islands in early response genes was observed in five additional human and mouse innate immune cells and human cell-line data sets with different stimuli (Additional file 2: Figure S9 and Additional file 4: Table S3).

Whilst we see consistent enrichment of short CpG islands amongst early response genes across a number of different cell types and stimuli, we also observed several exceptions to this pattern. For example, oestrogen receptor *α* (ER *α*) stimulated breast adenocarcinoma cells, and retinal pigment epithelial cells induced to an epithelial-to-mesenchymal transition with TGF *β* + TNF *α* displayed the opposite pattern of enrichment, i.e. longer CpG islands are enriched in early response genes (Fig. 4b). This opposing pattern of enrichment was also observed in mouse Cd8+ cytotoxic T cells stimulated with interferon *β* (IFN- *β*), amongst others (Additional file 2: Figure S10 and Additional file 4: Table S3). That we observe opposing patterns of enrichment within the same cell type but using different stimuli, i.e. MCF7 cells, indicates that there is a stimulus-specific mechanism. Previous work on stimuli-specific differences in IEG induction in mouse macrophages found a bias towards a SWI/SNF complex-dependence for IFN- *β* but not LPS [31]. The SWI/SNF complex in both mammals and yeast plays a role as an ATP-dependent nucleosome re-modeller [32, 33]. Steroid-hormone nuclear receptors, including ER *α*, require SWI/SNF complex proteins in the induction of target genes [34–37]. Likewise, induction of TGF- *β* responsive genes via Smad 2/3 in epithelial cells is dependent on the SWI/SNF ATPase BRG1 [38, 39]. Thus, the SWI/SNF-(in)dependence between different stimuli reconciles the discordance in CpG island size usage between stimuli, even within cells of the same type and origin (Fig. 4).

If short CpG islands are fast response elements and gene expression noise diminishes with CpG island size, we would expect to observe that both short and noisy promoters are up-regulated immediately post-stimulation. To corroborate our hypothesis that short and noisy CpG island promoters are responsive promoters, we used single-cell RNA-seq expression data from stimulated BMDCs [40]. Genes that are highly variable in unstimulated cells should be those genes most likely to respond. We performed differential expression testing between unstimulated BMDCs and those cells 1 hour post-LPS stimulation. Comparing genes that are up-regulated with their noise in unstimulated cells revealed that these genes are consistently more variable than those genes that are either down-regulated (one-tailed Kolmogorov–Smirnov test *p*=7.89×10^−123^) or for which expression does not change (one-tailed Kolmogorov–Smirnov test *p*=6.97×10^−10^). This effect is consistent for CpG island promoters up to 1.5 kb, but was still dependent on CpG island size (Fig. 4c). This relationship was maintained after adjusting for the mean dependence using *r*CV^2^ (Additional file 2: Figure S11b). Notably, we did not observe the same tendency for noisy genes with non-CpG island promoters to be immediately up-regulated (Additional file 2: Figure S11).

## Discussion and conclusions

In this study, we investigated a number of promoter features and their association with differences in gene expression noise. In concordance with a recent systematic analysis in mESCs [10], we find that CpG island promoters are less variable than their non-CpG island counterparts. This observation, and its generalisability to different cell types and mammalian species, implicates transcription regulatory mechanisms as important sources of expression noise [7–9, 41]. Further, the defining characteristics of CpG islands are associated with differences in noise. In particular, our finding that CpG island size is negatively correlated with expression noise indicates that variation in CpG island composition explains a proportion of the differential noise between promoters.

Based on these observations, we propose that short CpG islands, as noisy promoters, may be indicative of transcriptional dynamism. Our observation of short CpG island enrichment amongst early response genes indicates the potential role for promoter-associated short CpG islands to act as rapid transcriptional response elements. These findings, supported by evidence that highly variable genes in unstimulated BMDCs are up-regulated immediately upon stimulation, suggest a potential cost/benefit trade-off between stochastic transcriptional activation (noise) and how quickly a promoter is able to respond to an external stimulus. Interestingly, Antolovic et al. [42] recently showed that noisier genes in undifferentiated *Dictyostelium* were more likely to be up-regulated upon induction of a differentiation signal. These findings in a less complex eukaryote organism, which are concordant with our observations in BMDCs, highlight the potential for gene expression noise to mark genes primed to respond as a general feature of transcriptional regulation. *Dictyostelium* species lack experimentally validated CpG islands, which suggests that the exact mechanism by which gene expression noise arises may differ between phylogenetic clades or species. This is important, as we do not observe the same responsiveness for noisy non-CGI promoters (Additional file 2: Figure S11c). Transcriptional dynamics and promoter sensitivity to a modulating stimulus (repressive or activatory) are influenced by the chromatin landscape [43, 44] and the presence of paused RNA polymerase [45, 46]. The class of genes associated with immediate response (IEGs) are rapidly induced within a few minutes of stimulation, without the need for prior protein synthesis [30]. IEGs are associated with specific promoter architectures (high affinity TATA boxes and CpG islands) and encode shorter mRNA transcripts [30]. Induction of IEGs is associated with post-translational modification of histone proteins, such as lysine acetylation and histone H3 serine phosphorylation [44]. These modifications facilitate the binding of 14-3-3 and the SWI/SNF ATPase BRG1 [44]. Our observation that specific stimuli do not appear to utilise short CpG islands as IEGs is potentially reconciled by the stimulus-specific recruitment of SWI/SNF complex proteins to these IEGs [34–37, 44]. Moreover, the current lack of data on SWI/SNF complex dynamics in early response to stimuli suggests that there is the potential to discover the molecular mechanisms underlying these observations. Thus, our observation that short CpG islands are enriched amongst early response genes indicates a potentially novel mechanism for mammalian IEG induction.

Whilst we use CpG island size as a definition for these rapid response elements, there are most likely additional influences from static and dynamic promoter features. For example, recent work in *Drosophila* has highlighted the importance of promoter shape in transcriptional dynamics and the importance of noise [47]. CpG islands have been proposed as the vertebrate equivalent of polycomb response elements (PREs) in *Drosophila*, which is supported by evidence of PRC chromatin modification and PcG binding enrichment over these genomic features (reviewed in [48]). Thus, the link between CpG island size and response agility is potentially linked to differential dynamics in chromatin histone modifications or the binding of histone modification readers and writers.

Modelling of chromatin dynamics suggests that slow changes in chromatin modifications are required to induce transcriptional changes [49], a finding supported by the response model put forward by Klose et al. [50]. Berry et al. model the impact of robustness to *trans*-activation, supportive of a buffer in the responsive model, and note that the width of the *cis* memory window can have a drastic impact on responsiveness to noise in *trans* activator levels [49]. Thus, modelling of chromatin dynamics is concordant with the following: (a) CpG island size influences expression noise and (b) the smallest CpG islands provide the least buffering against stochastic fluctuations in *trans* activator levels.

The exact nature and source of gene expression noise within short CpG island promoters is not immediately clear. Recent evidence indicates that one common emerging source of gene expression noise is related to chromatin accessibility and dynamics [8–10]. It has been proposed that CpG islands generally have a more open or accessible conformation based on their lower affinity for histone proteins [31, 51]. However, more accessible chromatin is also associated with more promiscuous expression, which would not explain the increased noise observed at short CpG islands. Whether differences in noise arise due to the presence of paused or actively transcribing RNA polymerases, or the constitutive presence of *trans* activating factors requires further study.

Note that our measures of single-cell noise capture both technical and biological sources of variability. We do not expect technical sources of variation to distort systematically relationships between promoter architecture and gene expression noise, across multiple single-cell RNA-seq data sets. One possible source of confounding between our genomic features and noise is the sequence content of genic regions (e.g. coding sequence G + C content). However, exon G+C content is not related to CpG island composition or genomic position, indicating that our findings cannot be explained by technical sources of variation.

In this work, we used a mean-adjusted measure of noise, *r*CV^2^. We derive this measure by fitting a reciprocal relationship between CV^2^ and the mean expression for all genes, using a gamma distributed generalised linear model [12]. The extent to which this regression removes any relationship between mean expression and the features and contexts in which we test gene expression noise is dependent on the quality of this model fit. CV^2^ is a real-valued positive variable whose sampling distribution is likely to be similar to other variance-like statistics that are usually described by a *χ*^2^ distribution. The *χ*^2^ distribution is a special case of a gamma distribution; thus, CV^2^ can be appropriately described by a gamma distribution. Therefore, we suggest that modelling the relationship between the mean and gene expression noise using a gamma generalised linear model provides the most appropriate model in this context.

Our findings indicate that noisy genes, independent of mean expression, tend to be the most rapidly up-regulated. This suggests that many genes are poised to react to environmental stimuli which may have consequences for understanding how, as well as the speed with which a cell is primed to react to its environment. For instance, stress response genes have been identified as particularly noisy [9, 52] and the expression of stress response genes diverges between yeast species [53]. This indicates that highly responsive genes might evolve at different rates compared to more stably expressed and less noisy genes. Indeed, mutation accumulation experiments in yeast identified a correlation between the degree to which a gene promoter evolves and expression noise [54]. Fast-evolving genes, i.e. those whose expression is not constrained by stabilising selection, may also represent a mechanism for generating phenotypic heterogeneity in a population [53, 55]. This may further provide a way to either promote or buffer against multiple different perturbations, e.g. environmental or mutational. Whether these principles generalise to more complex eukaryote organisms remains to be seen, and provides an exciting possible avenue of research.

In conclusion, we have shown that short and noisy CpG islands may act as rapid response elements to external stimuli. These findings raise interesting questions about what role transcriptional variation has to play in the cellular and physiological response of organisms to their environment, and how these mechanisms have evolved.

## Methods

### Single-cell RNA-seq data processing

Where available, gene-by-cell-expression count matrices were downloaded from the Gene Expression Omnibus (GEO, https://www.ncbi.nlm.nih.gov/geo/) or ArrayExpress (https://www.ebi.ac.uk/arrayexpress/; see Additional file 3: Table S2 for a list of accessions) [14, 19, 40, 56, 57]. Cells with low count numbers (<100 000) or that had been flagged as poor quality by the study meta-data (where available) were removed prior to normalisation. Within each data set separately, genes that were expressed in <1*%* of cells were removed, prior to cell size factor normalisation using the deconvolution approach [58] and log2 transformation.

### Gene expression noise

Gene expression noise was calculated as the absolute residual squared coefficient of variation (*r*CV^2^) from the fit between the mean log2 expression and squared coefficient of variation (CV^2^), parametrised as in Brennecke et al. [12]. Specifically, CV^2^ was fitted using a gamma generalised linear model:

1 $$ E\left[\text{CV}_{i}^{2}\right] = 1 + \frac{1}{\mu_{i}},  $$

where

$$\mu_{i} = \log_{2} \left(\frac{1}{m} \sum^{m}_{j=1}{\frac{c_{ij}}{s_{j}}} \right), \quad i \in \{1, 2, \dots, m\}, $$

is the average expression of gene *i*, *c*_*ij*_ is the read count for gene *i* in cell *j* and *s*_*j*_ is the deconvolution size factor. The squared coefficient of variation is

$$\text{CV}_{i}^{2} = \frac{\hat{\sigma}_{i}^{2}}{\mu_{i}^{2}} $$

and $\hat {\sigma _{i}^{2}}$ is the sample variance for gene *i*. The gene expression noise, $r\text {CV}_{i}^{2}$, for gene *i*, is, therefore, calculated as the deviation of the expected value from the observed $\text {CV}_{i}^{2}$, i.e. $r\text {CV}_{i}^{2} = \left |\text {CV}_{i}^{2} - E\left [\text {CV}_{i}^{2}\right ]\right |$.

To test the influence of genomic features on gene expression noise formally, we fitted a linear model to *r*CV^2^ in each data set and tested the slope of the fitted line, i.e. the regression coefficients. We observed that *r*CV^2^ had a long-tailed distribution (Additional file 2: Figure S1c). Ordinary least squares regression compares the mean difference between values of a predictor variable on the response variable. The breakdown point of the mean is 0; thus, only a single extreme value is required to bias its estimate. The median has a breakdown of 0.5, that is, it requires more than half of the values to be outliers to bias its estimation. Thus, ordinary least squares regression would be inappropriate in this context where there are many extreme *r*CV^2^ values, whilst a robust linear regression that uses the median would not be susceptible to the same extreme outlier values.

### Genome annotations

Gene promoters were defined for each mm10 and hg19 gene based on the Ensembl build v86 annotations (https://www.ensembl.org/index.html). Promoter regions encompassed −1 kb and + 500 bp centred on the transcriptional start site (or beginning of the first annotated exon), accounting for strand. Total transcript lengths, exon length variation and number of exons were calculated from the Ensembl v86 General Transfer Format (GTF) file.

CpG island data were downloaded from the UCSC Genome browser [59] for hg19 and mm10 using the table browser tool [60]. Promoter sequence GC content was calculated for each promoter using bed2fasta from CGAT tools [61].

Transcription factor binding motifs were predicted over the length of all promoters using the MEME suite tool FIMO, with motif positional weight matrices provided from the JASPAR CORE vertebrate TF motif database (2016) [62, 63].

### ChIP sequencing data processing

ChIP-seq libraries derived from mESCs were downloaded from GEO accession GSE36114 [64]. Sequences were downloaded from the European Nucleotide Archive (ENA; https://www.ebi.ac.uk/ena). Known Illumina sequencing adaptors were removed using trimmomatic prior to alignment to mm10 using bwa-mem [65]. Aligned sequences were quantified over non-overlapping 200-nt windows on the mm10 genome in all ChIP and input libraries. log2-fold enrichment was calculated between each chromatin modification ChIP library and its matched input for each window. Signals over promoter intervals were calculated as the average log2-fold enrichment across overlapping windows for each replicate, then averaged across all replicates for the respective chromatin modification immunoprecipitation.

Bivalent promoters were calculated based on overlapping H3K27me3 and H3K4me3 ChIP signals. The H3K4me3 chromatin modification signal was dichotimised based on the local minimum of the kernel density estimate over all gene promoters. The H3K37me3 ChIP signals displayed a long tail without any noticeable minimum in density. Thus, to dichotomise the H3K27me3 signal, a threshold was set as the mean value across all gene promoters. Gene promoters were assigned to either *active* (H3K4me3) or *repressed* (H3K27me3) if the promoter signal exceeded 1.5 times the threshold for the relevant chromatin modification. Genes that exceeded both thresholds were assigned to the *bivalent* category.

### Defining mouse embryonic development genes

Mouse embryonic development genes for Additional file 2: Figure S6 were defined from J1 embryonic stem cells (ESCs) differentiated over 14 days and measured at 11 time points [66]. Gene expression data were downloaded from the StemBase data base (April 2018, http://www.stembase.ca) [67] and were based on measurements with an Affymetrix Mouse Expression 430a array. Embryonic genes were defined based on log2-fold change >0 over the differentiation time course, tested in a generalised linear model with a moderated *t*-test [68], at a false discovery rate of 1%.

### CAGE processing and time-series analysis

Peak annotations for a human time series from a cap analysis of gene expression with sequencing (CAGE-seq) were acquired from the FANTOM5 consortium website (http://fantom.gsc.riken.jp/5/data/) [69, 70]. Tag counts were downloaded and split by time-series experiment. CpG islands were assigned to peak annotations within 500 bp centred on each CAGE peak. Peaks expressed in ≤75*%* of samples were removed prior to analysis for each time-series data set separately. Moderated log2-fold changes between time points were calculated and tag counts were modelled using a negative binomial generalised linear model implemented in the Bioconductor package edgeR [71, 72].

### RNA-seq processing and time-series analysis

Gene-by-sample count matrices were downloaded from GEO for the relevant accessions [73, 74]. Genes containing on average >5 read counts across all samples were retained for analysis. Moderated log2-fold changes were estimated for each time-point comparison using the Bioconductor package DESeq2 [75].

### CpG island size enrichment testing

For each data set, we compared the proportion of genes, ranked by log2-fold change in expression between time points, that had a smaller CpG island overlapping the defined promoter region in the earliest time-point comparison. We then applied a one-tailed binomial test against a null hypothesis of a 50:50 relationship between rank and CpG island size. This process is analogous to gene set enrichment testing, using the ranked test statistics from a differential expression test, or equivalent to a paired sample sign test (Additional file 2: Figure S12).

### Code availability

The code used to perform the analyses and the process data are available from https://github.com/MarioniLab/CpGisland2017.

## Additional files

Additional file 1 Supplementary Table 1. CSV file describing genomic data sources and acquisition dates. (CSV 1 kb)

Additional file 2 Supplementary Figures. PDF of all supplementary figures S1–11. (PDF 3588 kb)

Additional file 3 Supplementary Table 2. CSV file describing single cell RNA-sequencing data sets used in this study, with cell numbers and accession numbers to publicly available data. (CSV 1 kb)

Additional file 4 Supplementary Table 3. CSV file of time-series gene expression data sets and CpG island size enrichment testing results. (CSV 1 kb)
