# Supplementary material for: CpG island composition differences are a source of gene expression noise indicative of promoter responsiveness
Source: Genome Biol. 2018 Jun 26;19:81. doi: 10.1186/s13059-018-1461-x (PMC6020341; doi:10.1186/s13059-018-1461-x)
Supplement: Supplementary file 2 — Supplementary Figures. PDF of all supplementary figures S1–11. (PDF 3588 kb) [file 13059_2018_1461_MOESM2_ESM.pdf]

CpG island composition differences are a source of gene expression noise indicative of promoter responsiveness: Supplementary Materials

Michael D. Morgan & John C. Marioni

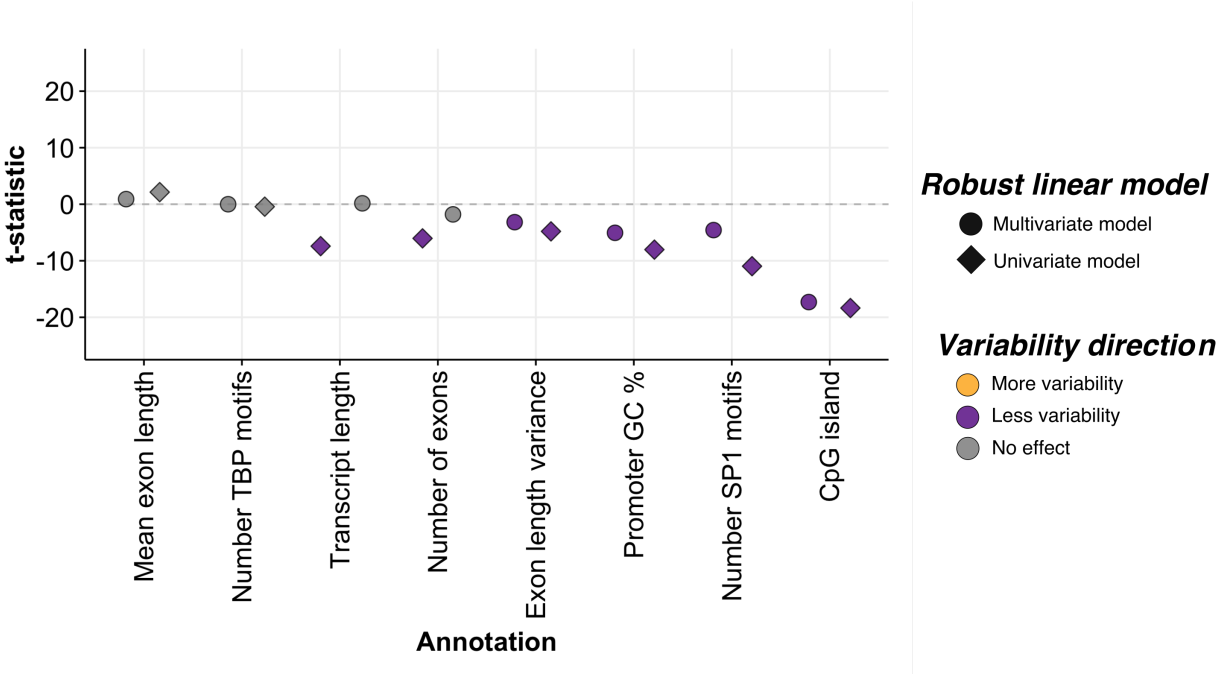

Supplementary Figure1: Cd4+ T cell gene expression variability models. Univariate (diamonds) and multivariate (circles) robust linear regression model t-test statistics (y-axis) are plotted for each genome feature tested for it's effect on gene expression variability. Orange filled symbols represent genomic features associated with increased gene expression variability, whilst purple filled symbols are features associated with lower expression variability. Symbols in grey do not show any statistical evidence for increased or decreased expression variability (P-value $<$ 0.05). The y-axis is restricted to the range [-50, 50] for clarity purposes.

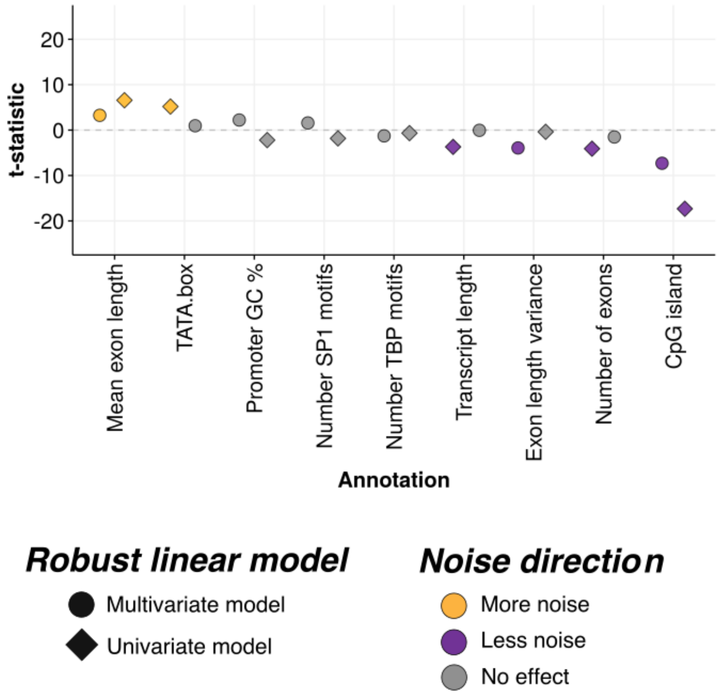

Supplementary Figure2: Mouse ESC gene expression variability models using EPDnew TATA-box promoter definition. Univariate (diamonds) and multivariate (circles) robust linear regression model t-test statistics (y-axis) are plotted for each genome feature tested for it's effect on gene expression variability. Orange filled symbols represent genomic features associated with increased gene expression variability, whilst purple filled symbols are features associated with lower expression variability. Symbols in grey do not show any statistical evidence for increased or decreased expression variability (P-value < 0.05). The y-axis is restricted to the range [-50, 50] for clarity purposes.

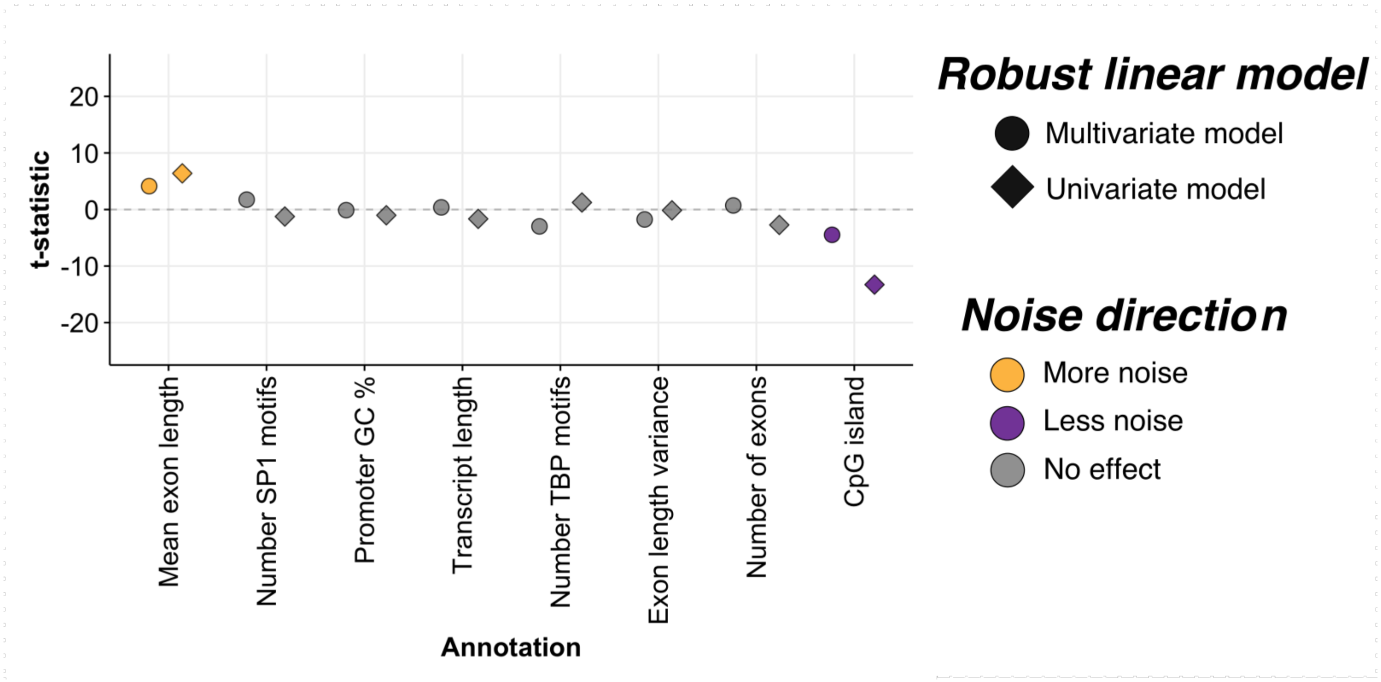

Supplementary Figure3: Associations between CpG islands and gene expression noise do not arise due to fragment duplication as a source of variation in single cell RNA sequencing experiments. Univariate (diamonds) and multivariate (circles) robust linear regression model t-test statistics (y-axis) are plotted for each genome feature tested for it's effect on gene expression variability calculated using transcript numbers estimated from mESCs using UMIs. Orange filled symbols represent genomic features associated with increased gene expression variability, whilst purple filled symbols are features associated with lower expression variability. Symbols in grey do not show any statistical evidence for increased or decreased expression variability (P-value < 0.05). The y-axis is restricted to the range [-50, 50] for clarity purposes.

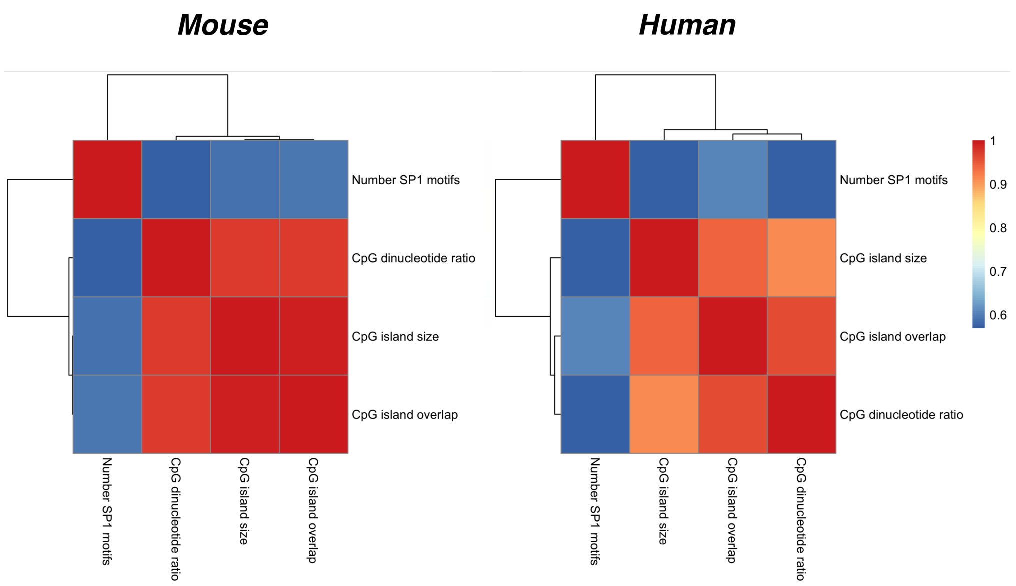

Supplementary Figure4: CpG island features are highly correlated with each other. Spearman rank correlations between CpG island features - island size (kbp), overlap with gene promoter (number of nucleotides), and ratio of observed CpG dinucleotides to the expected based on the number of G+C nucleotides, normalized by island length.

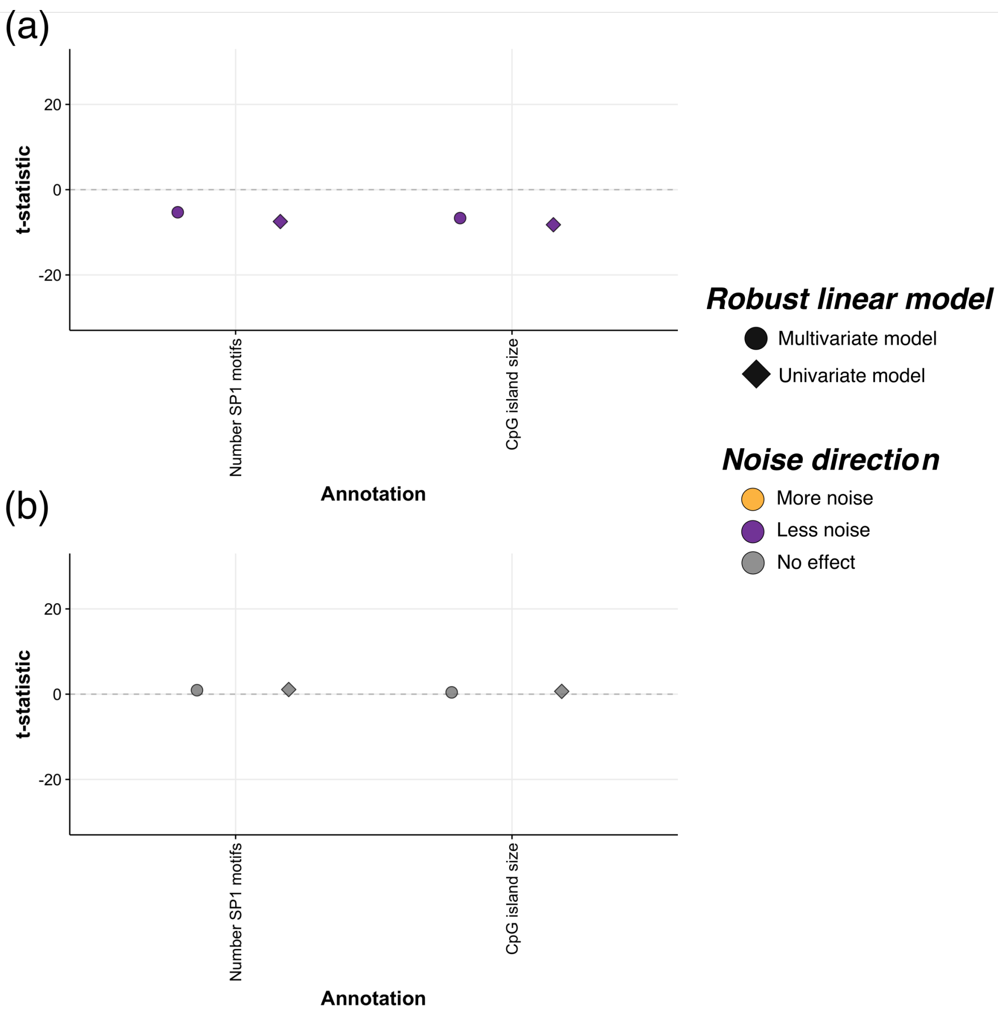

Supplementary Figure5: CpG island feature gene expression variability robust linear models for (a) human pancreatic α-islet and (b) human pancreatic β-islet cells. Univariate (diamonds) and multivariate (circles) robust linear regression model t-test statistics (y-axis) are plotted for each genome feature tested for it's effect on gene expression variability. Orange filled symbols represent genomic features associated with increased gene expression variability, whilst purple filled symbols are features associated with lower expression variability. Symbols in grey do not show any statistical evidence for increased or decreased expression variability (P-value < 0.05). The y-axis is restricted for clarity purposes.

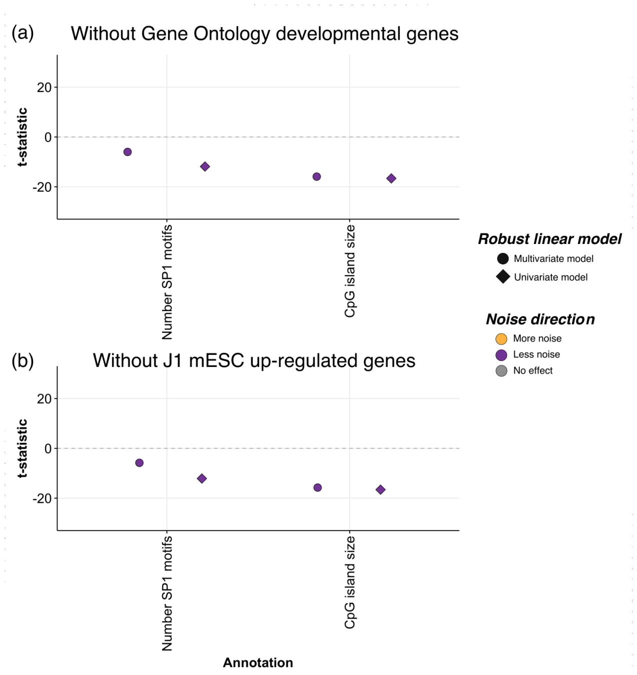

Supplementary Figure6: Developmental genes do not drive the relationship between CpG island features and gene expression variability in mouse ESCs. CpG island features were regressed on $r\text{CV}^2$ individually (univariate; diamonds) or simultaneously (multivariate; circles), whilst excluding all genes either (a) annotated to the gene ontology terms *embryo development*, *multicellular organism development*, *anatomical structure development* and *developmental process* (n=994), or (b) genes that are significantly up-regulated in J1 mESCs over 14 days of differentiation (n=2121). Orange filled symbols represent genomic features associated with increased gene expression variability of the associated genes, whilst purple filled symbols are features associated with lower expression variability. Symbols in grey do not show any statistical evidence for increased or decreased expression variability (P-value < 0.05). The y-axis is restricted to the range [-50, 50] for clarity.

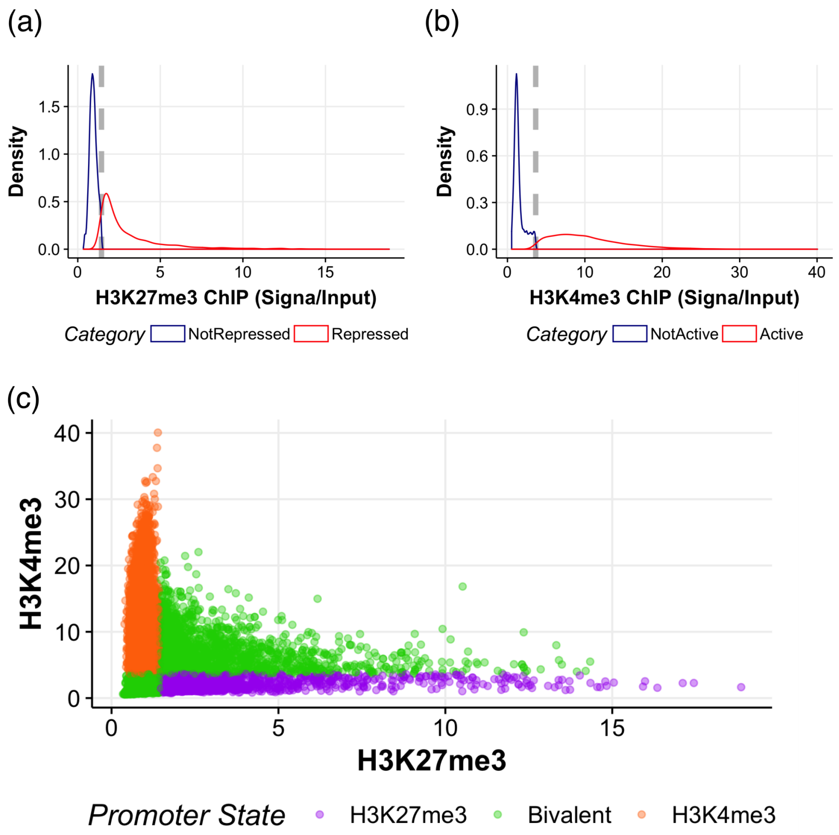

Supplementary Figure7: Categorisation of promoters into *Repressed*, *Bivalent* or *Active* based on the combined signal of H3K27me3 and H3K4me3 ChIP enrichment. (a) Kernel density plot demonstrating the threshold of H3K27me3 signal (ChIP/input) to classify promoters into *Repressed* or *NotRepressed*, which is denoted by the dashed grey line. (b) The categorisation of promoters into *Active* and *NotActive* based on the relative signal of H3K4me3 over the gene promoters; the grey line denotes the decision threshold. (c) Genes which are simultaneously classified into *NotRepressed* **and** *NotActive*, or are simultaneously classified into *Repressed* **and** *Active* are assigned to the *Bivalent* group (green points).

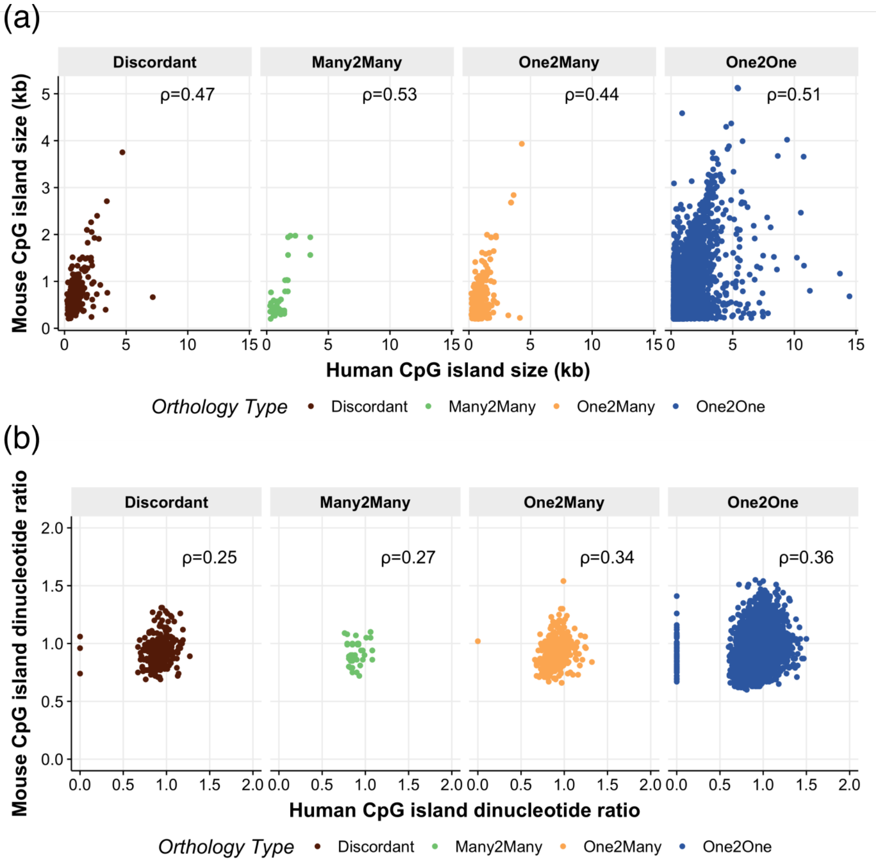

Supplementary Figure8: Correlation between mouse and human promoter CpG islands, split by Ensembl homology type (*Discordant* indicates a disagreement between the homology type for mouse:human and human:mouse mappings). (a) CpG island size relationship, between human (x-axis) and mouse (y-axis), and (b) CpG island CpG observed/expected ratio , i.e. CpG dinucleotide density, between human (x-axis) and mouse (y-axis). Spearman rank correlation (ρ) is denoted on each panel.

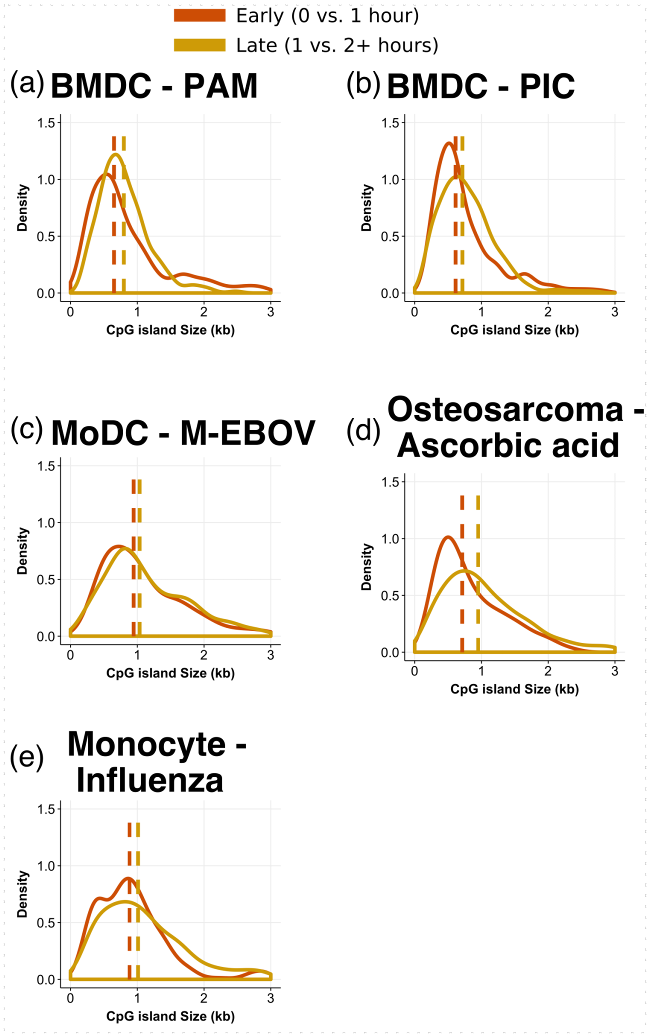

Supplementary Figure9: Short CpG island enrichment in early response genes in mouse bone marrow-derived DCs stimulated with PAM (a) or PIC (b). Additional enrichments are observed in mouse monocyte derived DCs stimulated with Ebola glycoprotein (EBOV) (c) Osteosarcoma cell line, Saos-2, stimulated with ascorbic acid (d) as well as human monocytes exposed to influenza virus (e). Axis denoting CpG island size (density plots x-axis, box plot y-axis) are truncated at 3kb for clarity. Binomial test p-values for each stimulus are shown in Supplementary Table 3.

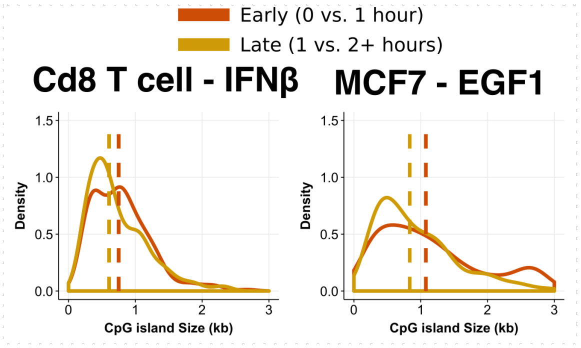

Supplementary Figure10: Longer CpG island enrichment in early response genes in *in vitro*-derived Cd8+ cytotoxic T cells stimulated with interferon β (IFN-β), and breast adenocarcinoma cell line, MCF-7, stimulated with EGF1 (b). Axis denoting CpG island size (density plots x-axis, box plot y-axis) are truncated at 3kb for clarity.

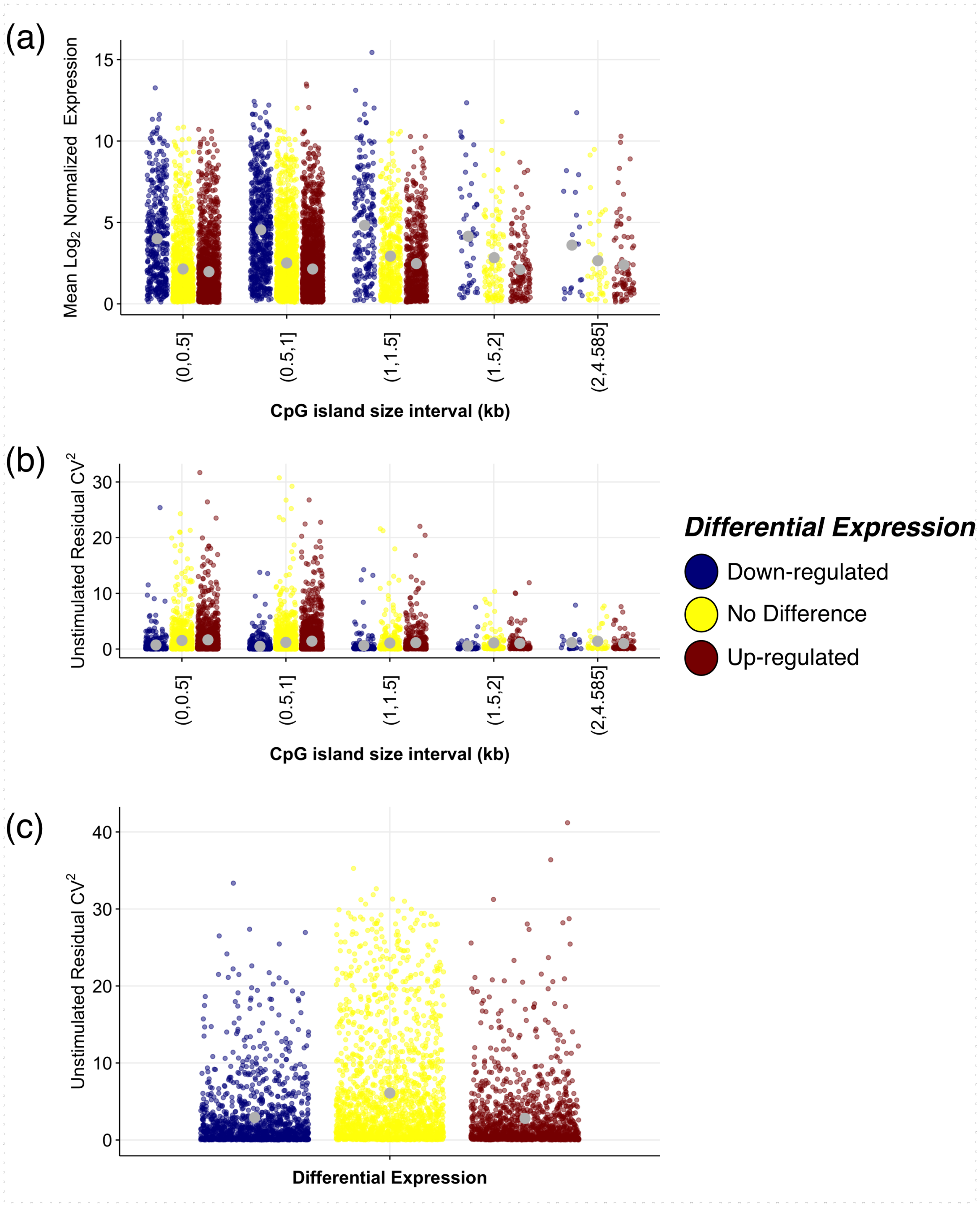

Supplementary Figure11: (a) Single cell BMDC mean $\log_2$ normalized gene expression partitioned between CpG islands size, and split into differential expression category based on whether the gene is up-regulated, down-regulate, or unchanged following LPS stimulation. (b) Residual CV^2 for CpG island genes in single unstimulated BMDCs, partitioned into CpG island sizes and DE category. (c) Residual CV^2 for non-CpG island genes in single unstimulated BMDCs, partitioned into DE categories. Grey filled circles in each plot represent the mean value for that category for the relevant gene expression summary statistic.

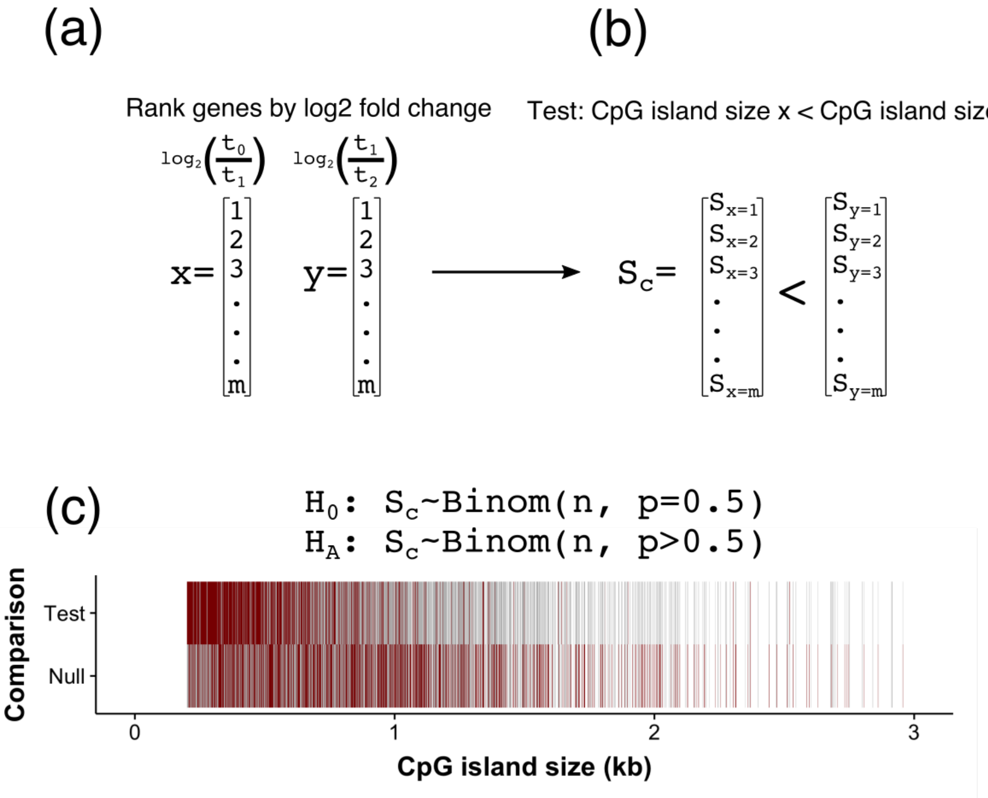

Supplementary Figure12: CpG island size enrichment testing schematic. (a) Genes are ranked on the $\log_2$ fold change between time points for the two time points to be compared. (b) The ranked lists x and y are used to compare the CpG island sizes for these ranked gene list, and calculate whether the CpG island size in x is smaller than the equal rank in y, denoted S_c. (c) Under the null hypothesis of no enrichment for short CpG islands, S_c is expected to be binomially distributed with a probability 0.5. An example enrichment is demonstrated in the heatmap, where there is a clear enrichment of short CpG islands relative to the expectation under the null hypothesis.
